# Sex-Differential Association Between Serum Uric Acid and Incident Hypertension in a Chinese Population With Normal Body Mass Index: A Prospective Observational Study

**DOI:** 10.31083/RCM42700

**Published:** 2025-10-14

**Authors:** Yanglie Ye, Minjun Yang, Mengmeng Shao, Shengjie Wu, Xiaoyun Wu

**Affiliations:** ^1^Department of Emergency, The First Affiliated Hospital of Wenzhou Medical University, 325000 Wenzhou, Zhejiang, China; ^2^Department of Cardiology, Taizhou Hospital of Zhejiang Province Affiliated to Wenzhou Medical University, 317000 Linhai, Zhejiang, China; ^3^Department of Rehabilitation, The First Affiliated Hospital of Wenzhou Medical University, 325000 Wenzhou, Zhejiang, China; ^4^Department of Cardiology, The First Affiliated Hospital of Wenzhou Medical University, 325000 Wenzhou, Zhejiang, China

**Keywords:** sex, serum uric acid, hypertension, body mass index

## Abstract

**Background:**

Increasing evidence suggests a positive correlation between serum uric acid (SUA) levels and incident hypertension (IHT). However, few studies have focused on the sex-differential impact of SUA levels on IHT in populations with a normal body mass index (BMI).

**Methods:**

This study included participants without hypertension who had a BMI in the normal range (18.5–23.9 kg/m^2^). Sex-specific quartiles of SUA levels (Q1–Q4) were defined as: ≤180, 181–213, 214–249, and >249 μmol/L for females; ≤282, 283–324, 325–373, and >373 μmol/L for males. IHT was considered present when systolic blood pressure (SBP) was ≥140 mmHg or diastolic blood pressure (DBP) was ≥90 mmHg, or antihypertensive drugs were used. Cox proportional hazards models and mediation analysis were performed to estimate hazard ratios (HRs) and potential mediators in the relationship between sex-differential SUA levels and IHT.

**Results:**

This study included 24,538 participants, comprising 13,063 females and 11,475 males, with an IHT of 4.9% in females and 11.4% in males during 24 (12, 36) months. In the sex-stratified analysis, females exhibited higher unadjusted HRs for Q4 versus Q1 (HR = 3.487, 95% CI: 2.701–4.500; *p* < 0.001) compared to males (HR = 2.016, 95% CI: 1.719–2.365; *p* < 0.001). After adjustment for multiple variables, the HRs for females remained higher than those for males (2.237 [1.670–2.998] vs. 1.904 [1.601–2.265]); however, the magnitude of the difference was notably reduced. Mediation analysis indicated that the association between SUA levels and IHT was primarily driven by age (19.42%), low-density lipoprotein (LDL) cholesterol (10.90%), and triglycerides (10.46%) in females, and by BMI (9.94%), triglycerides (TG) (8.73%), serum creatinine (7.26%), and age (7.23%) in males.

**Conclusion:**

SUA levels among Chinese adults with a normal BMI range were positively associated with IHT, with an apparent stronger association in females than in males.

## 1. Introduction 

Hypertension, characterized by elevated blood pressure levels, is a major global public health concern due to its association with an increased risk of cardiovascular diseases, stroke, and renal complications [1, 2, 3]. Despite significant advancements in preventive and therapeutic approaches, the prevalence of hypertension remains high, contributing substantially to morbidity and mortality worldwide [4]. While various factors influence the development of hypertension, the role of serum uric acid (SUA) levels in this context has received considerable attention in recent years.

As a metabolic end product of purine metabolism, SUA has been implicated in the pathogenesis of hypertension through several mechanisms. Elevated SUA levels have been associated with endothelial dysfunction, oxidative stress, inflammation, and activation of the renin-angiotensin-aldosterone system, all of which contribute to the development and progression of hypertension [5, 6]. Moreover, SUA has been shown to promote vascular smooth muscle cell proliferation and arterial stiffness, further exacerbating hypertension [7]. However, the relationship between SUA and hypertension appears to be complex, influenced by various factors including sex and body mass index (BMI).

Sex-based differences in the relationship between SUA and hypertension have become increasingly evident, with studies suggesting that the strength and nature of this association may vary by sex [8, 9, 10]. Additionally, the impact of BMI on this association has attracted attention, given the rising prevalence of obesity worldwide [11]. Despite these observations, studies investigating the sex-differential association between SUA and incident hypertension (IHT) in individuals with normal BMI are limited, necessitating further exploration to elucidate potential sex-specific differences in this relationship.

The purpose of this study was to investigate the sex-differential association between SUA levels and the incidence of hypertension in a Chinese population with normal body mass index, with particular attention to potential sex-specific differences. Furthermore, we sought to explore potential mechanisms underlying any observed sex-specific differences, thereby contributing to our understanding of the pathophysiology of hypertension and paving the way for sex-tailored approaches in the management of hypertension.

## 2. Materials and Methods

### 2.1 Study Design and Population

This retrospective study involved participants from the First Affiliated Hospital of Wenzhou Medical University, who underwent annual health checkups between December 2009 and 2014. Participants included in the study were those without a previous hypertension diagnosis or antihypertensive drug use, had a BMI within the normal range (18.5–23.9 kg/m^2^) at the time of their initial examination, and had attended at least one follow-up assessment. This study was conducted in accordance with the Declaration of Helsinki. Personal identifiers were replaced with health examination numbers to ensure confidentiality. The study’s protocol received approval from the Ethics Committee of the First Affiliated Hospital of Wenzhou Medical University (KY2025-R205).

### 2.2 Measurements and Definitions

Before the health checkup, participants were required to fast and avoid smoking and strenuous activity for at least 12 hours. The examination included a doctor’s physical evaluation, anthropometric data collection, blood pressure (BP) measurements, and blood sampling. Height was measured without shoes to the nearest centimeter, and weight was recorded in light clothing, also without shoes, to the nearest 0.1 kg. BMI was calculated in the conventional manner as body mass divided by the square of body height. Measurements of systolic and diastolic BP were obtained with a noninvasive automated sphygmomanometer (OMRON, Kyoto, Japan), following a 5-minute rest period with the participant seated in a calm environment. Duplicate measurements were performed during the same appointment, with the mean value considered for subsequent analyses. If the initial readings varied by ≥10 mmHg, additional measurements were made, and the analysis used the average of the last two measurements. Levels of SUA, fasting plasma glucose (FPG), triglycerides (TG), high-density lipoprotein cholesterol (HDL-C), low-density lipoprotein cholesterol (LDL-C), creatinine (Cr), alanine aminotransferase (ALT), aspartate aminotransferase (AST), hemoglobin (HB), platelet (PLT), and white blood cells (WBC) were determined.

### 2.3 Follow-up

The occurrence of hypertension was tracked over time through yearly follow-up examinations conducted throughout the duration of the study. These follow-up evaluations mirrored the baseline examination procedures. IHT was defined by meeting one or more of the following criteria: (1) elevated BP ≥140/90 mmHg at examination; (2) commencement of antihypertensive treatment; or (3) a confirmed hypertension diagnosis documented by a physician during annual follow-up.

### 2.4 Statistical Analysis

Given the pronounced sex-related differences in SUA distribution, participants were grouped into sex-specific SUA quartiles: for females—Q1: ≤180, Q2: 181–213, Q3: 214–249, Q4: >249 µmol/L; and for males—Q1: ≤282, Q2: 283–324, Q3: 325–373, Q4: >373 µmol/L. Continuous variables were described using mean ± SD or median (interquartile range, IQR), depending on distribution. Categorical variables were reported as counts and percentages. Differences were assessed using independent sample *t*-tests for continuous variables and chi-squared tests for categorical variables. Cox proportional hazards models estimated hazard ratios (HRs) and 95% confidence intervals (CIs) for the risk of IHT across SUA level quartiles. The cumulative incidence of IHT during follow-up was estimated using Kaplan–Meier analysis. To examine potential nonlinear trends between SUA and the risk of hypertension, generalized additive models (GAMs) were employed separately for males and females. Stratified analyses explored the impact of pre-specified factors (age, BP, BMI, Cr, and FPG) on the SUA-hypertension relationship. To further explore the mechanisms underlying the association between SUA levels and IHT, we performed mediation analysis to estimate the indirect effects of potential mediators. The analysis quantified the proportion of the total effect of SUA on hypertension that could be explained by intermediate variables such as age, BP, BMI, Cr, and FPG. The mediation effect was evaluated using a regression-based approach with bootstrapping to estimate confidence intervals for indirect effects. Proportion mediated was defined as the proportion of the total effect mediated via the mediator. Separate mediation models were constructed for males and females to account for potential sex-specific pathways. Least absolute shrinkage and selection operator (LASSO) regression introduces an L1 regularization term to ordinary least squares regression, effectively shrinking certain coefficients toward zero. This method serves the dual purpose of feature selection and preventing model overfitting, thereby enhancing the model’s interpretability and generalizability. It is particularly useful in scenarios with numerous correlated predictors, helping to pinpoint the most significant features impacting the outcome. Data management and analyses were performed using SPSS Statistics version 24.0 (SPSS, IBM Corp., Armonk, NY, USA) and the statistical package R version 4.3.2 (R Foundation for Statistical Computing, Vienna, Austria). All tests were two-sided, with *p* values < 0.05 denoting statistical significance.

## 3. Result

### 3.1 Characteristics of Participants

The study comprised 24,538 participants, with baseline characteristics detailed by sex (Table 1). Among them, 13,063 were females, with an average age of 37.9 ± 11.0 years, and 11,475 were males, with an average age of 39.6 ± 13.4 years. SUA levels averaged 217.6 ± 54.8 µmol/L in females and 330.5 ± 70.9 µmol/L in males. Males showed significantly higher values than females in several parameters, including systolic blood pressure (SBP), diastolic blood pressure (DBP), BMI, age, SUA, HB, ALT, AST, serum creatinine (SCr), FPG, TG, LDL-C, and WBC. Conversely, HDL-C and PLT were significantly lower in females. During the study, 635 females (4.9%) and 1310 males (11.4%) developed hypertension during a follow-up of 24 (12, 36) months.

**Table 1.** **Baseline characteristics**.

Characteristics	Total (n = 24,538)	Women (n = 13,063)	Men (n = 11,475)	*p*-value
Incident hypertension	1945 (7.9%)	635 (4.9%)	1310 (11.4%)	<0.001
Age, years	38.7 ± 12.2	37.9 ± 11.0	39.6 ± 13.4	<0.001
BMI, kg/m^2^	21.3 ± 1.5	20.9 ± 1.5	21.8 ± 1.5	<0.001
SBP, mmHg	114.1 ± 9.4	111.5 ± 9.5	117.1 ± 8.4	<0.001
DBP, mmHg	70.4 ± 6.7	69.0 ± 6.5	72.0 ± 6.6	<0.001
Uric acid, µmol/L	270.4 ± 84.4	217.6 ± 54.8	330.5 ± 70.9	<0.001
ALT, U/L	19.3 ± 21.6	15.4 ± 11.6	23.5 ± 28.0	<0.001
AST, U/L	21.6 ± 10.6	20.0 ± 8.6	23.3 ± 12.0	<0.001
Serum Cr, µmol/L	79.2 ± 18.3	67.1 ± 9.2	93.0 ± 16.3	<0.001
FPG, mmol/L	5.1 ± 0.7	5.0 ± 0.5	5.2 ± 0.9	<0.001
TG, mmol/L	1.2 ± 1.0	1.0 ± 0.5	1.5 ± 1.3	<0.001
HDL-C, mmol/L	1.5 ± 0.3	1.6 ± 0.3	1.3 ± 0.3	<0.001
LDL-C, mmol/L	2.4 ± 0.6	2.3 ± 0.6	2.5 ± 0.6	<0.001
HB, g/L	137.4 ± 14.7	126.9 ± 10.2	148.5 ± 9.9	<0.001
PLT, 10^9^/L	189.9 ± 44.0	195.1 ± 45.5	184.0 ± 41.4	<0.001
WBC, 10^12^/L	5.9 ± 1.5	5.7 ± 1.4	6.2 ± 1.5	<0.001

ALT, alanine aminotransferase; AST, aspartate aminotransferase; BMI, body mass index; Cr, creatinine; DBP, diastolic blood pressure; FPG, fasting plasma glucose; HB, hemoglobin; HDL-C, high-density lipoprotein cholesterol; LDL-C, low-density lipoprotein cholesterol; PLT, platelet count; SBP, systolic blood pressure; TG, triglyceride; WBC, white blood cell.

### 3.2 Association Between Uric Acid and Incident Hypertension

**Fig. 1.**
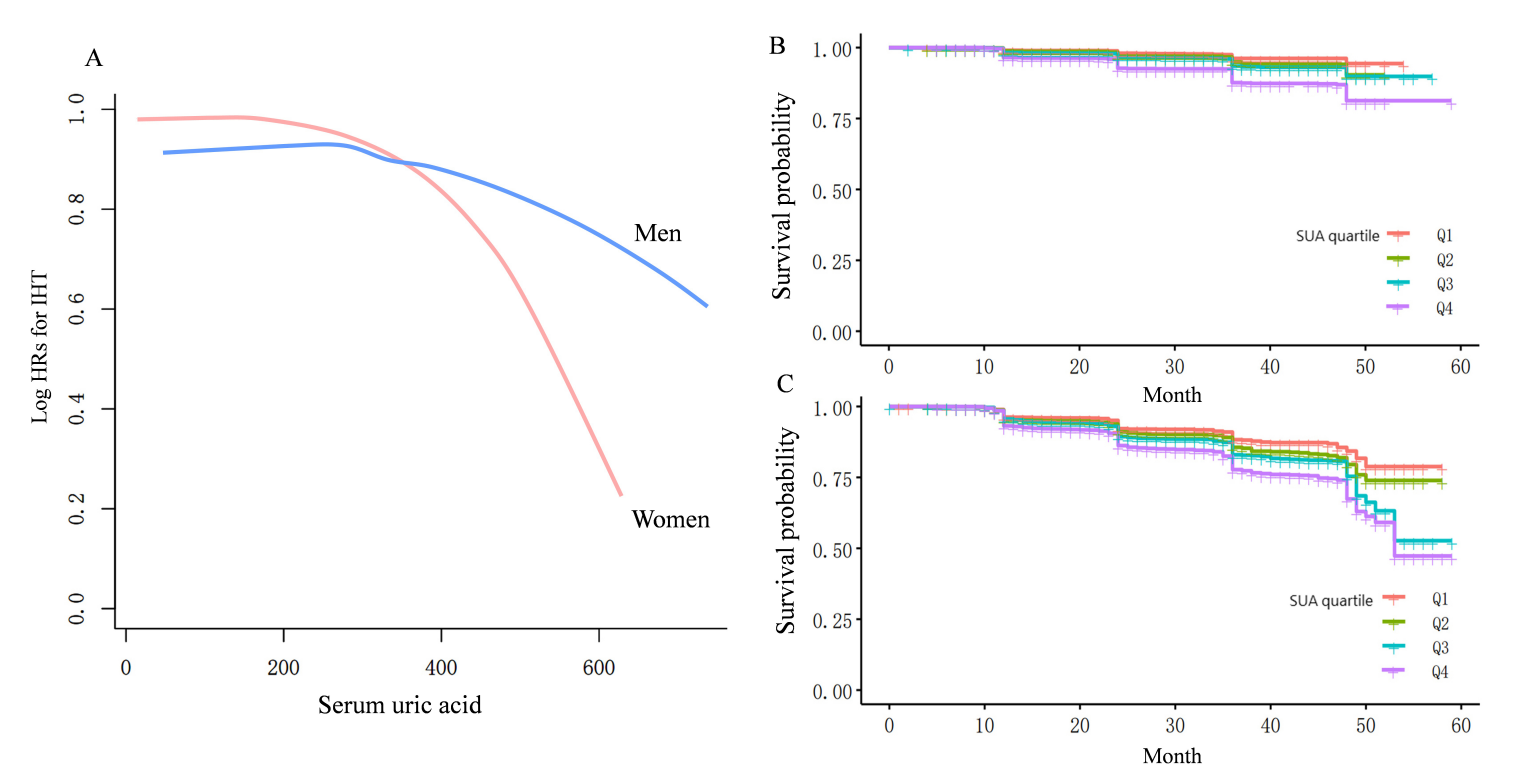
**Curve-fitting and Kaplan–Meier analysis of hypertension incidence and sex-specific serum uric acid levels**. (A) The curve-fitting analysis indicates that the risk of hypertension associated with increased serum uric acid levels is significantly higher in females than in males. (B,C) Kaplan–Meier analysis reveals that elevated serum uric acid levels substantially increase the risk of developing hypertension for both genders, with females experiencing a greater increase in hypertension incidence compared to Q1. HRs, hazard ratios; IHT, incident hypertension; SUA, serum uric acid.

Fig. 1 indicates that uric acid levels have a more substantial impact on the incidence of hypertension in females compared to males. As SUA levels increase, there is a more marked rise in the incidence of hypertension among females, suggesting a stronger association between elevated uric acid levels and the development of hypertension in this group.

**Fig. 2.**
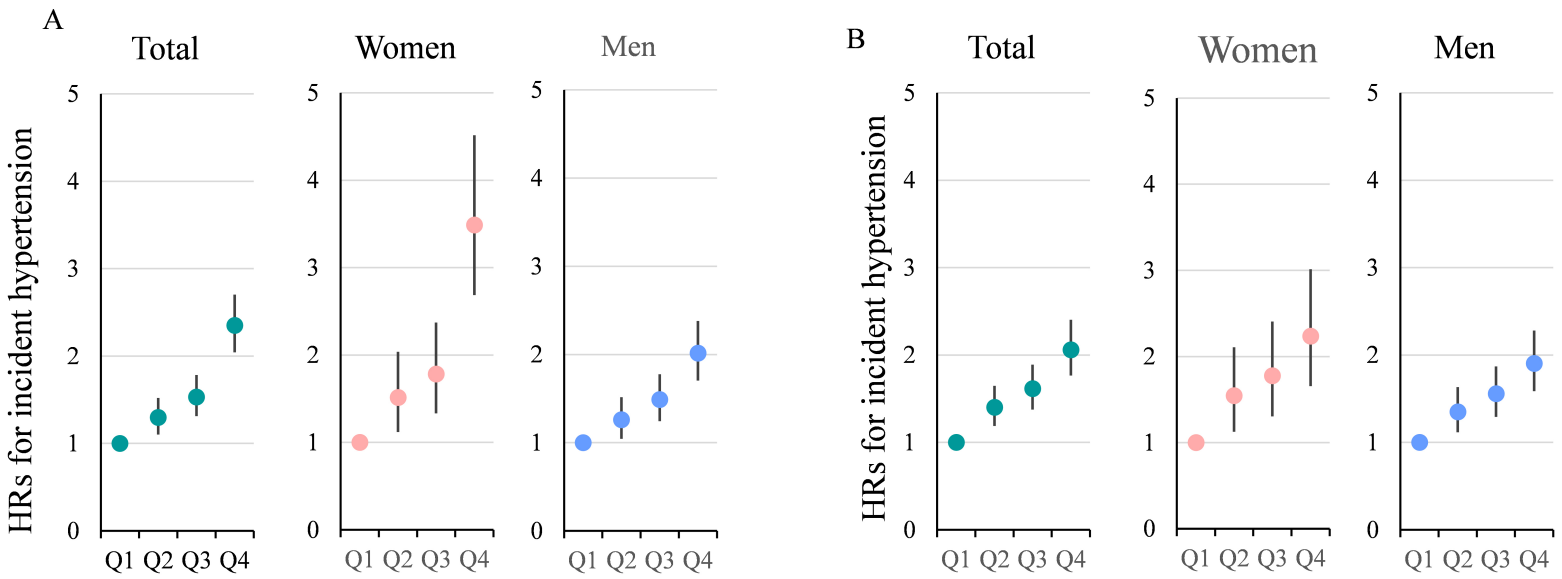
**Hazard ratios (HRs) with 95% confidence intervals (CIs) for quartiles of serum uric acid levels stratified by Sex**. (A) unadjusted HRs; (B) shows HRs adjusted for all known factors. These factors include age, body mass index, systolic blood pressure, diastolic blood pressure, fasting plasma glucose, triglycerides, high-density lipoprotein cholesterol, low-density lipoprotein cholesterol, serum creatinine, alanine aminotransferase, aspartate aminotransferase, hemoglobin, platelet count, and white blood cell count. It is evident that, before adjustment, the HRs for females in Q4 vs Q1 are significantly higher than those for males. After adjustment for multiple variables, the HRs for females remain higher than those for males, but the magnitude of the difference is notably reduced.

**Fig. 3.**
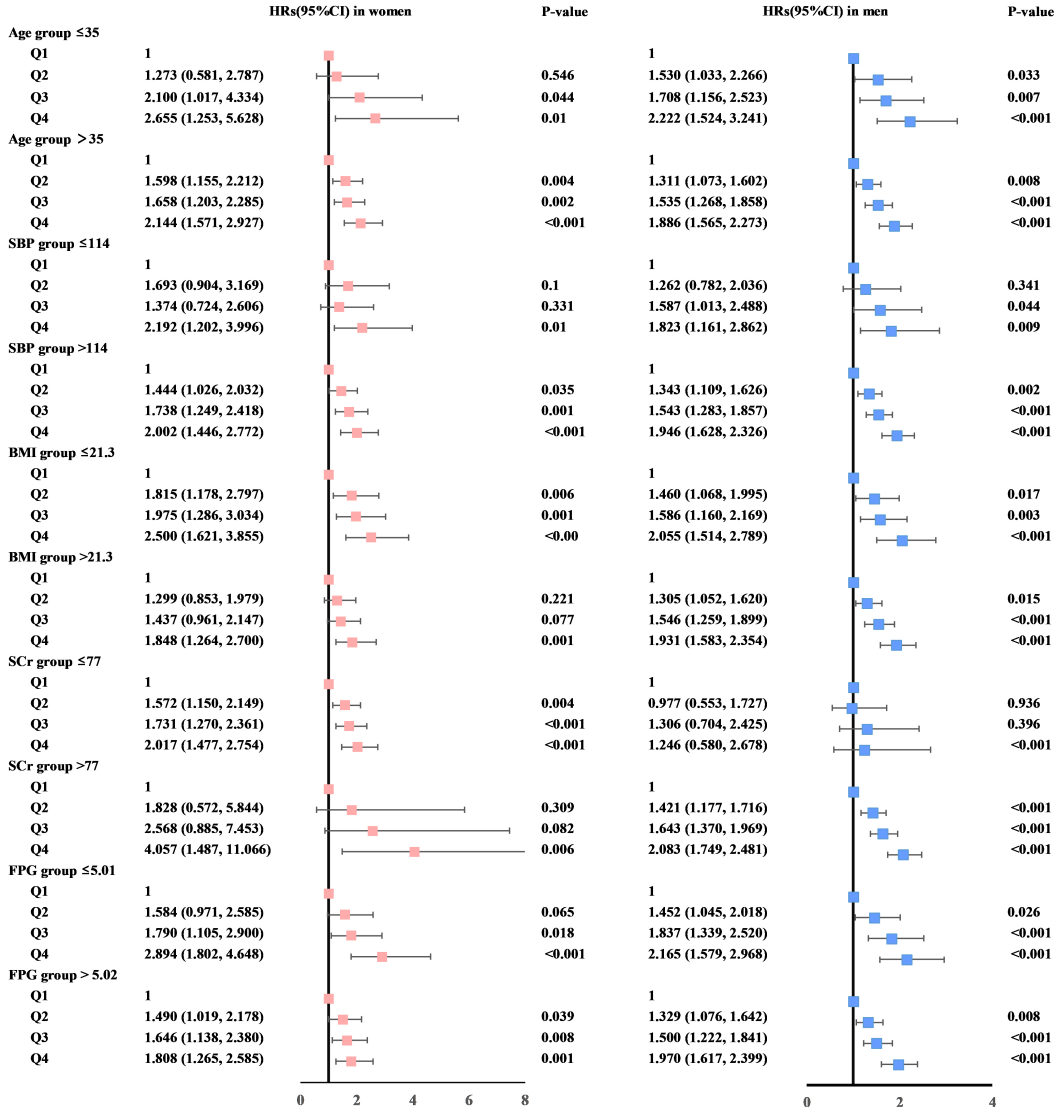
**Forest plots display sex-stratified hazard ratios (HRs) with 95% confidence intervals (CIs) for serum uric acid quartiles, adjusted for multiple covariates**. These factors include alanine aminotransferase, aspartate aminotransferase, age, body mass index (BMI), diastolic blood pressure, fasting plasma glucose (FPG), hemoglobin, high-density lipoprotein cholesterol, low-density lipoprotein cholesterol, platelet count, serum creatinine (SCr), systolic blood pressure (SBP), triglycerides, and white blood cell count. This detailed adjustment aims to isolate the effect of serum uric acid (SUA) levels on the risk of hypertension, accounting for a wide array of potential confounders.

Cox regression results showing IHT risk by SUA quartiles are presented in Fig. 2. In the total population, the unadjusted HRs for IHT in Q4 versus Q1 was 2.351 (95% CI: 2.056–2.688, *p* < 0.001). After adjusting for known risk factors, the association remained significant but was attenuated, with an HR of 2.061 (95% CI: 1.779–2.388, *p* < 0.001). Sex-stratified analysis revealed that the unadjusted HR for Q4 versus Q1 was higher in females (3.487; 95% CI: 2.701–4.500; *p* < 0.001) than in males (2.016; 95% CI: 1.719–2.365; *p* < 0.001). After adjustment for multiple variables, the HRs for females remained higher than those for males (2.237 [1.670–2.998] vs. 1.904 [1.601–2.265]); however, the magnitude of the difference was notably reduced. The relationship between baseline SUA and IHT was further evaluated in stratified analyses by key clinical factors such as age, BP, BMI, SCr, and FPG. The findings consistently indicated a positive association between the highest category of SUA levels and an increased risk of developing hypertension. These results are illustrated in forest plots of HRs presented in Fig. 3. Interaction analysis was performed to assess the consistency of these associations across the subgroups defined by the aforementioned factors. The analysis revealed that the positive correlations between elevated SUA levels and the risk of hypertension were uniformly observed across all stratified groups, with all of interaction *p* values > 0.05. These consistent findings indicate that the link between elevated SUA and the risk of hypertension remains stable across different clinical and demographic subgroups.

**Fig. 4.**
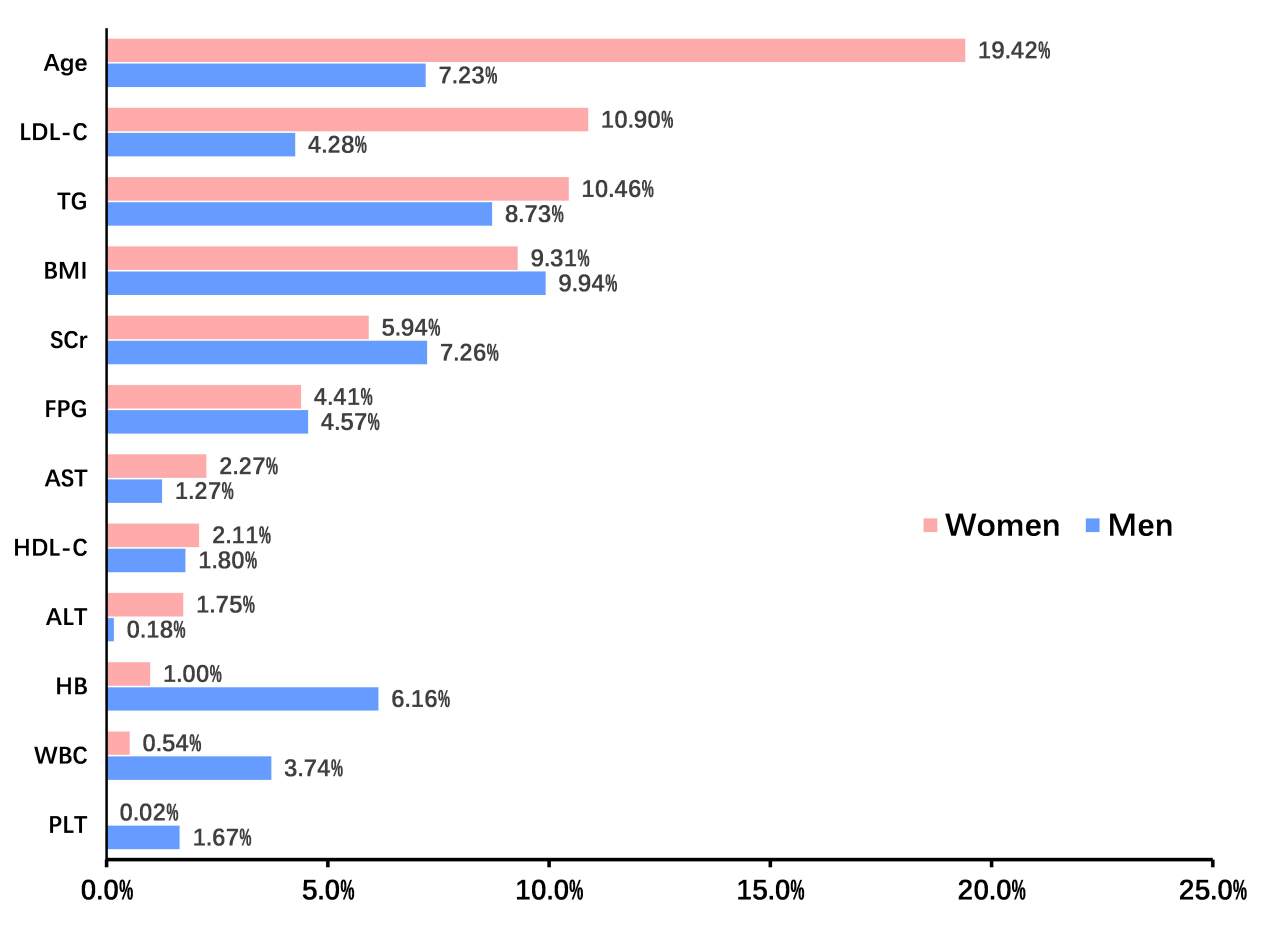
**Mediation analysis identified indirect effects of covariates on this relationship between serum uric acid and incident hypertension in females and males**. ALT, alanine aminotransferase; AST, aspartate aminotransferase; BMI, body mass index; FPG, fasting plasma glucose; HB, hemoglobin; HDL, high-density lipoprotein; LDL, low-density lipoprotein; PLT, platelet count; TG, triglyceride; WBC, white blood cell; SCr, serum creatinine.

The mediation analysis was performed to elucidate how various covariates mediate the link between elevated SUA levels and the development of hypertension, with separate analyses for females and males. Fig. 4 shows that the association between SUA levels and IHT was primarily driven by age (19.42%), LDL-C (10.90%), and TG (10.46%) in females, and by BMI (9.94%), TG (8.73%), SCr (7.26%), and age (7.23%) in males.

### 3.3 Development of a Risk Prediction Model for Incident Hypertension

**Fig. 5.**
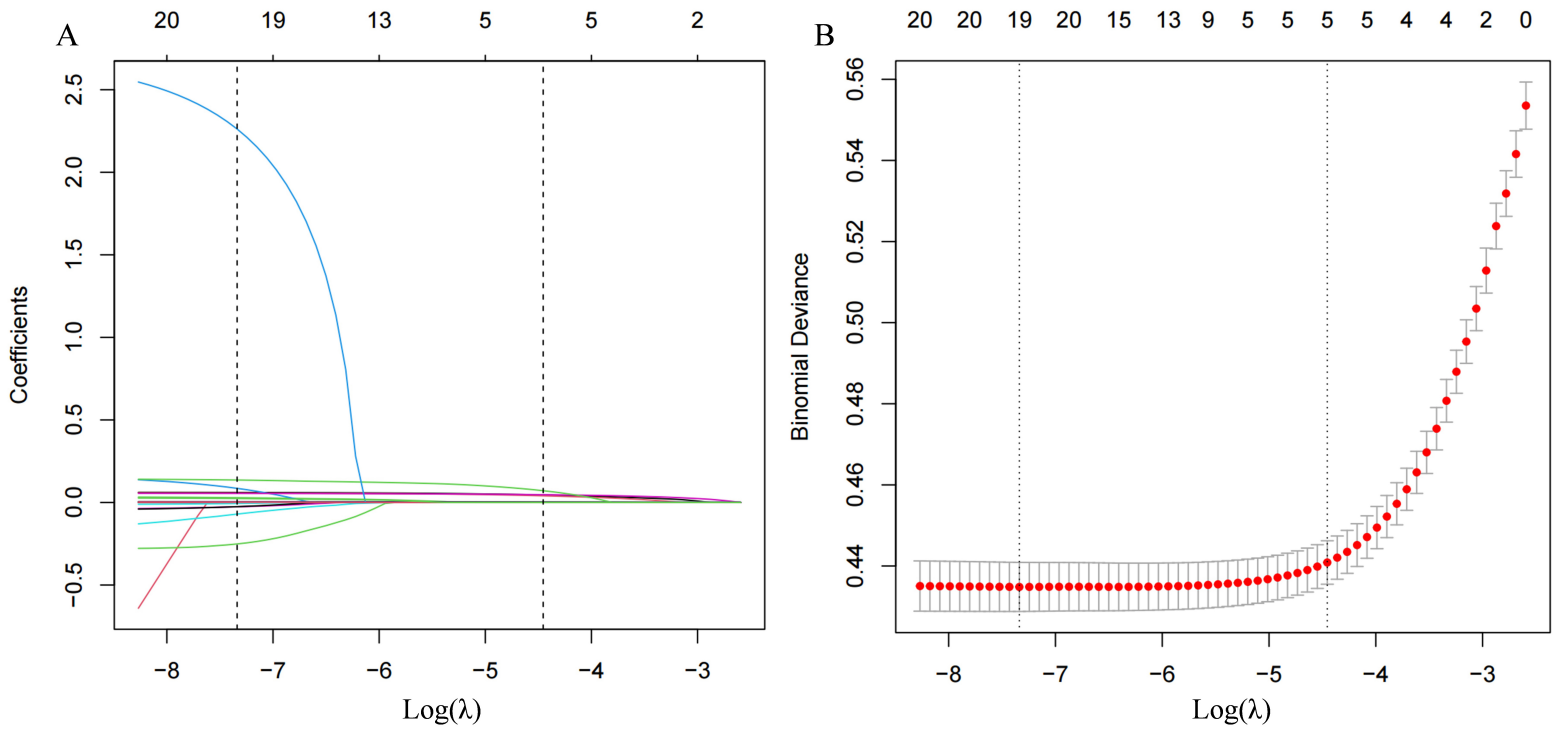
**The LASSO penalized regression analysis for identifying key dietary factors related to incident hypertension**. (A) This part illustrates the coefficient shrinkage process for all 15 covariates involved in the study. The graphical representation showcases how coefficients for different dietary factors adjust under varying degrees of shrinkage, with each line’s color denoting a distinct feature. (B) Displays a 10-fold cross-validation of the LASSO regression model, a technique that ensures the model’s reliability and predictive accuracy by dividing the dataset into ten parts to validate the model ten times on different subsets. LASSO, least absolute shrinkage and selection operator.

**Fig. 6.**
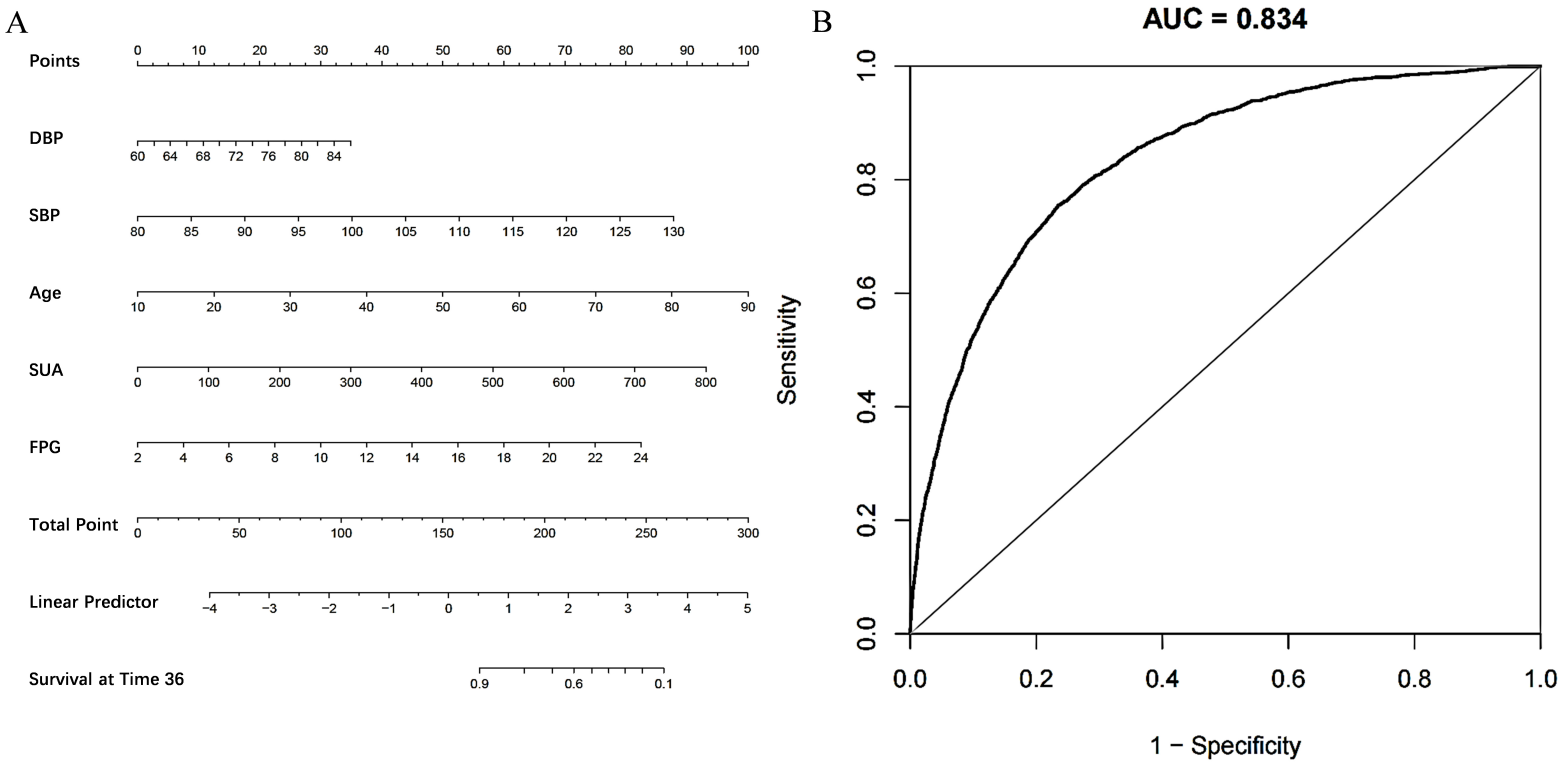
**Establishment and validation of a risk prediction model for incident hypertension**. (A) A nomogram model was constructed from five critical factors pinpointed through LASSO regression analysis. This visual tool enables the calculation of an individual’s risk of developing hypertension by aligning each of the identified factors along their respective axes. (B) Features the receiver operating characteristic (ROC) curve used to assess the nomogram model’s predictive accuracy for hypertension. LASSO, least absolute shrinkage and selection operator; AUC, area under the curve; DBP, diastolic blood pressure; SBP, systolic blood pressure; SUA, serum uric acid; FPG, fasting plasma glucose.

The study employed a LASSO penalized regression model to identify key parameters closely associated with the incidence of hypertension, as illustrated in Fig. 5. The LASSO model identified DBP, SBP, age, SUA, and FPG as the factors most strongly correlated with the risk of developing hypertension. Based on these findings, a risk prediction nomogram was developed, incorporating these five factors due to their substantial predictive value for the incidence of hypertension. The effectiveness of this nomogram model in predicting the risk of hypertension was further validated by a receiver operating characteristic (ROC) curve, demonstrating considerable predictive accuracy with an area under the curve (AUC) of 0.834, as shown in Fig. 6.

## 4. Discussion

This study reveals a significant sex-specific correlation between SUA levels and hypertension incidence among Chinese adults maintaining a normal BMI, thereby enhancing our understanding of hypertension’s pathophysiology. Our research adds to the accumulating evidence that elevated SUA levels constitute a significant risk factor for hypertension, with the association appearing stronger in females than in males.

A previous longitudinal study conducted over an average follow-up period of 5.41 years in a Taiwanese cohort indicated 1119 persons (34.3%) had experienced progression to a higher blood pressure stage and 496 persons (15.2%) had developed hypertension. The adjusted HRs comparing the highest and lowest SUA quartiles were 1.68 (1.23–2.04) for IHT [12]. Recent data from the Health Examinees Study, a community-based prospective cohort study conducted in Korea from 2004 to 2013 demonstrated that higher baseline SUA levels were associated with greater increases in BP during follow-up, and this effect was strongest in females aged 40–49 years (β = 0.87 and *p* < 0.01 for systolic blood pressure) [13]. Aligned with these prior studies, our findings confirm a distinct positive relationship between SUA levels and the likelihood of hypertension, persisting even after accounting for various confounders. This relationship was notably more pronounced among females, especially within the highest SUA quartile, suggesting a potential sex-specific vulnerability to the hypertensive consequences of uric acid. These observations are consistent with research conducted across diverse populations, reinforcing the global significance of SUA as an indicator for the risk of hypertension [5, 6].

In our study, the risk of IHT became statistically significant from the second SUA quartile (181–213 µmol/L in females and 283–324 µmol/L in males), suggesting that even modestly elevated uric acid levels—below the conventional hyperuricemia threshold—may carry clinical relevance. This is consistent with the URRAH study [14], which proposed cardiovascular-specific SUA cut-offs as low as 5.6 mg/dL (≈333 µmol/L) for cardiovascular mortality and 4.7 mg/dL (≈279 µmol/L) for all-cause mortality. These values are significantly lower than the traditional definition of hyperuricemia. The similarity of our findings with those of URRAH underscores the importance of establishing cardiovascular-specific cut-off points for SUA, rather than relying solely on thresholds used for gout or nephrolithiasis. Such tailored thresholds may improve early identification of at-risk individuals, especially in populations with normal weight and among females, and could enhance the precision of cardiovascular risk stratification [3, 15].

The sex-specific effects of SUA on hypertension, as delineated in our research, also suggest underlying biological and possibly lifestyle-driven disparities in the mechanisms of hypertension between genders. Existing literature indicates that estrogen may offer a protective buffer against urate-induced hypertension in premenopausal females, which could elucidate the increased sensitivity noted after menopause [16, 17]. Moreover, variations in crucial mediators such as LDL-C, TG, and BMI underscore the presence of gender-specific routes through which uric acid influences blood pressure [18, 19]. These insights underline the importance of adopting gender-tailored strategies in the prevention and treatment of hypertension, especially for individuals presenting with high SUA levels.

Overweight has traditionally been a foundational metric in evaluating cardiovascular risk, supported by substantial evidence associating obesity with various health complications, notably hypertension [20]. Nonetheless, our study highlights a significant oversight in this conventional approach by demonstrating that individuals with a normal BMI range can still be susceptible to hypertension if they have elevated SUA levels. This finding points to the inadequacies of using only normal or high BMI classification exclusively as an indicator of cardiovascular health and makes a compelling case for including metabolic markers like SUA in risk assessment protocols. The contribution of BMI to cardiovascular risk assessment is nuanced, reflecting the realization that while obesity remains a pivotal risk factor for hypertension, metabolic factors within normal weight ranges indicate a complex relationship between body composition, metabolic health, and hypertension [21]. A study aimed to examine cardiometabolic health misclassifications given standard BMI categories found that nearly half of overweight individuals, 29% of obese individuals, and even 16% of obesity type 2/3 individuals were metabolically healthy [22]. In the present study, even after adjustment for multiple variables, the HRs for Q4 versus Q1 was 2.061 (1.779, 2.388). The pronounced link between elevated SUA levels and the risk of hypertension within individuals of a normal BMI underscores the need for a paradigm shift in how cardiovascular risk is assessed. It elevates the role of SUA from a secondary marker to a primary factor in identifying individuals at heightened risk for hypertension.

Additionally, our mediation analysis identified that the association between SUA levels and hypertension was primarily driven by age (19.42%), LDL cholesterol (10.90%), and triglycerides (10.46%) in females, and by BMI (9.94%), TG (8.73%), serum creatinine (7.26%), and age (7.23%) in males. In females, the effect of uric acid on the development of hypertension appears to be more strongly mediated by age. The difference between the two groups may be partly attributed to hormonal influences. This hormonal protection might explain the increased sensitivity to hypertension observed in females post-menopause, as the decline in estrogen levels could diminish this protective effect, thereby heightening the risk associated with elevated SUA [23]. These mediators underscore the multifaceted nature of the development of hypertension, implicating lipid metabolism and body composition as well as uric acid as critical factors. The role of these mediators suggests potential intervention points, including lipid management and weight control, to mitigate the risk of hypertension in individuals with elevated SUA levels. Future research should aim to elucidate the mechanisms by which these factors interact with uric acid, offering insights into more comprehensive and targeted prevention strategies.

Finally, while our study focused on SUA levels, it is important to recognize that the pathogenic role of uric acid may depend on its compartmentalization. Evidence suggests that intracellular uric acid can act as a pro-oxidant, promoting oxidative stress, endothelial dysfunction, and inflammation—mechanisms implicated in hypertension, insulin resistance, the metabolic syndrome, chronic kidney disease, and cardiovascular disease [5, 24]. Conversely, extracellular uric acid may exert antioxidant effects in the plasma but can contribute to gout and nephrolithiasis when present in excess amounts. Although we did not measure intracellular uric acid levels in this study, these mechanistic insights provide a broader context for understanding the complex role of uric acid in cardiometabolic diseases.

## 5. Limitations and Future Directions

While our study provides valuable insights, it is not without limitations. The observational nature of the study precludes definitive conclusions about causality between SUA levels and hypertension. Baseline data on the use of lipid-lowering or anti-diabetic medications were unavailable, which may have affected the analysis of certain metabolic parameters. Additionally, our findings are based on a Chinese population with normal BMI, and thus, may not be generalizable to other ethnic groups or populations with different BMI ranges. Future studies employing longitudinal designs and including diverse populations are warranted to validate our findings and explore the mechanisms underlying the observed associations with sex. Moreover, further research is needed to evaluate the effectiveness of interventions targeting SUA levels in the prevention of hypertension and to determine whether such interventions should be tailored by sex. Exploring the genetic basis of the variability in SUA levels and its relationship with hypertension could also offer new avenues for personalized medicine.

## 6. Conclusion

In conclusion, our study identifies the sex-differential association between SUA levels and the incidence of hypertension in a Chinese population with normal BMI, underscoring the potential of SUA as a predictive biomarker for the risk of hypertension. The stronger association observed in females highlights the importance of considering sex-specific factors in the assessment and management of hypertension. These findings advocate for a more nuanced approach to cardiovascular risk assessment, incorporating the monitoring of SUA levels alongside traditional risk factors. Further research into the biological mechanisms driving these associations and into effective, sex-specific intervention strategies is essential for advancing hypertension prevention and management.

## Data Availability

The datasets used during the current study are available from the corresponding author on reasonable request.

## Abbreviations

ALT, alanine aminotransferase; AST, aspartate aminotransferase; AUC, area under the curve; BMI, body mass index; Cr, creatinine; DBP, diastolic blood pressure; FPG, fasting plasma glucose; HB, hemoglobin; HDL-C, high-density lipoprotein cholesterol; HRs, hazard ratios; IHT, incident hypertension; LASSO, least absolute shrinkage and selection operator; LDL-C, low-density lipoprotein cholesterol; PLT, platelet count; ROC, receiver operating characteristic; SBP, systolic blood pressure; TG, triglyceride; WBC, white blood cell.
